# Simulation of Neutron/Self-Emitted Gamma Attenuation and Effects of Silane Surface Treatment on Mechanical and Wear Resistance Properties of Sm_2_O_3_/UHMWPE Composites

**DOI:** 10.3390/polym13193390

**Published:** 2021-10-02

**Authors:** Donruedee Toyen, Yupadee Paopun, Dararat Changjan, Ekachai Wimolmala, Sithipong Mahathanabodee, Theerasarn Pianpanit, Thitisorn Anekratmontree, Kiadtisak Saenboonruang

**Affiliations:** 1 Scientific Equipment and Research Division, Kasetsart University Research and Development Institute (KURDI), Kasetsart University, Bangkok 10900, Thailand; rdiddt@ku.ac.th (D.T.); rdiydp@ku.ac.th (Y.P.); rdidrc@ku.ac.th (D.C.); 2 Polymer PROcessing and Flow (P-PROF) Research Group, Division of Materials Technology, School of Energy, Environment and Materials, King Mongkut’s University of Technology Thonburi, Bangkok 10140, Thailand; ekachai.wim@kmutt.ac.th; 3 Department of Production Engineering, Faculty of Engineering, King Mongkut’s University of Technology North Bangkok, Bangkok 10800, Thailand; sithipong.m@eng.kmutnb.ac.th; 4 Department of Applied Radiation and Isotopes, Faculty of Science, Kasetsart University, Bangkok 10900, Thailand; fscitap@ku.ac.th (T.P.); thitisorn.an@ku.th (T.A.); 5 Specialized Center of Rubber and Polymer Materials in Agriculture and Industry (RPM), Faculty of Science, Kasetsart University, Bangkok 10900, Thailand

**Keywords:** UHMWPE, Sm_2_O_3_, neutron, gamma, silane coupling agent, wear resistance, mechanical properties, simulation

## Abstract

This work reports on the simulated neutron and self-emitted gamma attenuation of ultra-high-molecular-weight polyethylene (UHMWPE) composites containing varying Sm_2_O_3_ contents in the range 0–50 wt.%, using a simulation code, namely MCNP-PHITS. The neutron energy investigated was 0.025 eV (thermal neutrons), and the gamma energies were 0.334, 0.712, and 0.737 MeV. The results indicated that the abilities to attenuate thermal neutrons and gamma rays were noticeably enhanced with the addition of Sm_2_O_3_, as seen by the increases in µ_m_ and µ, and the decrease in HVL. By comparing the simulated neutron-shielding results from this work with those from a commercial 5%-borated PE, the recommended Sm_2_O_3_ content that attenuated thermal neutrons with equal efficiency to the commercial product was 11–13 wt.%. Furthermore, to practically improve surface compatibility between Sm_2_O_3_ and the UHMWPE matrix and, subsequently, the overall wear/mechanical properties of the composites, a silane coupling agent (KBE903) was used to treat the surfaces of Sm_2_O_3_ particles prior to the preparation of the Sm_2_O_3_/UHMWPE composites. The experimental results showed that the treatment of Sm_2_O_3_ particles with 5–10 pph KBE903 led to greater enhancements in the wear resistance and mechanical properties of the 25 wt.% Sm_2_O_3_/UHMWPE composites, evidenced by lower specific wear rates and lower coefficients of friction, as well as higher tensile strength, elongation at break, and surface hardness, compared to those without surface treatment and those treated with 20 pph KBE903. In conclusion, the overall results suggested that the addition of Sm_2_O_3_ in the UHMWPE composites enhanced abilities to attenuate not only thermal neutrons but also gamma rays emitted after the neutron absorption by Sm, while the silane surface treatment of Sm_2_O_3_, using KBE903, considerably improved the processability, wear resistance, and strength of the composites.

## 1. Introduction

Neutron technologies have been fast developed and widely utilized in different applications, including neutron imaging for material characterization and medical diagnostics [1,2,3], boron neutron capture therapy (BNCT) for cancer therapy [4], neutron moisture gauge for soil water measurement [5], and gemstone irradiation for color modification [6]. Although the mentioned benefits have substantially raised our overall quality of life, possibly excessive exposure to both chronic and acute neutron radiations could harmfully affect the health of users and the general public that may lead to severe diseases or death [7].

To reduce and/or limit the risks of excessive neutron exposure, appropriate and sufficient neutron-shielding equipment must be strictly utilized and applied in all nuclear facilities, as one of the three protective measures in radiation safety [8]. In principle, hydrogen-rich materials, such as natural rubber (NR), polyethylene (PE), concrete, and paraffin [9,10,11], are suitable and commonly used in neutron protection, as incident neutrons could scatter off hydrogen atoms and lose a relatively large portion of their initial kinetic energies, as shown in Equation (1):

(1) $$E_{k}=\frac{4Mm_{n}}{{\left(M+m_{n}\right)}^{2}}cos^{2}\beta$$

where *E*_k_, *M*, *m*_n_, and β are the kinetic energy losses of the neutron after the scattering, the recoil nucleus mass, the neutron mass, and the recoil angle of the nucleus, respectively. However, despite their acceptable attenuation abilities, it is still possible to further enhance the shielding abilities of the materials by introducing elements or compounds consisting of high-neutron-absorption cross-section isotopes, such as pure boron (B), B_2_O_3_, and B_4_C to the materials [9,10], by which the neutrons would be more efficiently absorbed and attenuated compared to those relying solely on neutron scattering, resulting in less materials required and potentially superior mechanical strength.

Recently, materials containing rare-earth oxides, especially Sm_2_O_3_, have gained great attention from researchers and product developers to replace typical boron compounds with samarium (Sm) that has an almost 8-fold higher neutron absorption cross-section (σ_abs_) than that of B (σ_abs_ of Sm and B are 5922 and 767 barns, respectively) [12], resulting in superior neutron-shielding capabilities compared to common borated materials at the same filler content. Furthermore, Sm and Sm_2_O_3_ have relatively higher atomic number (Z) and high density (*ρ*) (Z = 62 and *ρ* = 8.35 g/cm^3^, respectively) than B and B-containing compounds, making the former suitable for self-attenuation of gamma rays emitted after the neutrons have been absorbed by the Sm. This dual-shielding property of Sm_2_O_3_ for both neutrons and gamma rays has resulted in a simpler material design and remarkable shielding performance, which are crucially useful and important for workers in proximity to nuclear facilities with a photon-neutron-mixed environment [13]. Examples of Sm_2_O_3_-containing materials used in neutron protection are Sm_2_O_3_/UHMWPE composites [14], Sm_2_O_3_/Portland cement pastes [15], xPbO-(99-x)B_2_O_3_-Sm_2_O_3_ glass system [16], and carbon-fiber reinforced Sm_2_O_3_/polyimide composites [17], for which their neutron-shielding properties were found to be considerably higher than for borated materials, determined at the same filler content.

Among the mentioned examples, Sm_2_O_3_/UHMWPE composites are one of the most promising materials as they offer not only hydrogen-rich properties but also other preferable properties, such as exhibiting high impact and tensile strengths, excellent abrasion resistance, and a low frictional coefficient, making them suitable for use as fuel storage, casks for neutron transportation, movable partitions, and extruded profiles in nuclear facilities [18]. Recently, our previous work investigated the potential of applying Sm_2_O_3_/UHMWPE composites as neutron-shielding materials by determining their shielding, mechanical, electrical, and physical properties. The main results revealed that the addition of Sm_2_O_3_ in UHMWPE composites noticeably enhanced abilities to attenuate neutrons, as the values of the half-value layer (HVL) decreased from 248.0 mm in a neat UHMWPE to just 3.1 mm in samples with 50 wt.% Sm_2_O_3_ [14]. This enhanced the neutron-shielding properties of the Sm_2_O_3_/UHMWPE composites and substantially reduced the required material’s thickness and the costs associated with manufacturing, construction, and transportation of the shielding materials.

Nonetheless, although adding higher Sm_2_O_3_ contents to UHWMPE composites typically resulted in higher overall shielding properties, the poor surface compatibility between Sm_2_O_3_ and UHMWPE, as well as agglomerations of Sm_2_O_3_ particles, led to possible voids and non-uniform filler distribution in the matrix, resulting in reduced overall mechanical properties. These undesirable effects were evidenced by the decreases in tensile strength and elongation at break from 25.9 MPa and 1058% in a neat UHMWPE to 20.0 MPa and 117% in the samples containing 50 wt.% Sm_2_O_3_, respectively [14]. Furthermore, another report by Cao et al. indicated similar effects from having high Sm_2_O_3_ contents in UHMWPE composites, as the tensile strength and surface hardness (Shore D) decreased from ~22.8 MPa and ~84, respectively, in a neat UHWMPE sample to ~17.0 MPa and ~76, respectively, in a sample with 50 wt.% Sm_2_O_3_ [19]. This reduced the mechanical properties of the Sm_2_O_3_/UHMWPE composites and subsequently limited the durability and usability of the materials in actual applications.

To improve the surface compatibility, filler distribution, and wear/mechanical properties of the composites, a silane coupling agent could be used to treat surfaces of the filler, prior to further processes. The benefits of applying a silane coupling agent were emphasized in the report by Zhao et al., which showed that the surface treatment of nano-TiO_2_ with 3-aminopropyltrimethoxysilane and 3-isocyanatopropyltrimethoxysilane by an aqueous process led to a significant reduction in particle hydrodynamic diameters and the polydispersity index, which enhanced the particle dispersion stability of the nano-TiO_2_ during sample preparation [20]. In addition, Arslan and Dogan investigated the effects of three different silane coupling agents, namely (3-aminopropyl) triethoxysilane, (3-glycidyloxypropyl) trimethoxysilane, and (3-trimethoxysilyl) propylmethacrylate, on the mechanical properties of the basalt fiber (BF) reinforced poly (butyleneterefthalate) (PBT) composites, which indicated that all treated BF/PBT composites had higher overall mechanical properties than the non-treated BF/PBT composites, as shown by the increases in tensile strength, flexural strength, and impact strength from 47.6 MPa, 95.3 MPa, and 20.0 kJ/m^2^, respectively, in non-treated BF/PBT composites to 61.9–63.4 MPa, 97.8–106.4 MPa, and 20.5–24.0 kJ/m^2^, respectively, in treated BF/PBT composites [21]. These examples clearly suggested the advantages of utilizing appropriate silane coupling agents to improve filler distribution and the mechanical properties of the composites, which would be useful for use in radiation protection that normally contained high filler contents.

This work determined the simulated neutron and self-emitted gamma-shielding properties of Sm_2_O_3_/UHMWPE composites, using Monte Carlo coding (Particle and Heavy Ion Transport Code System (PHITS)) [22], for which the Sm_2_O_3_ contents varied in the range 0–50 wt.%, in 5 wt.% increments, and the results were independently verified by using a web-based program, namely XCOM [23]. The neutron energy investigated in this work was 0.025 eV for thermal neutrons, and the gamma energies were 0.334, 0.712, and 0.737 MeV (the energies of emitted gamma rays after neutron absorption by Sm). Shielding properties of interest were the linear attenuation coefficient (µ), the mass attenuation coefficient (µ_m_), and the half-value layer (HVL). In addition, the recommended Sm_2_O_3_ contents in UHMWPE composites for actual use were determined by comparing the simulated neutron-shielding properties with those from a commercial 5% borated material. Furthermore, this work experimentally determined the effects of a surface treatment of Sm_2_O_3_, using a silane coupling agent (3-aminopropyltriethoxysilane; KBE903), on elemental and chemical composition, morphological, wear resistance, frictional, and mechanical properties of UHMWPE composites that contained 25 wt.% Sm_2_O_3_. The KBE903 contents used for the surface treatment of Sm_2_O_3_ were varied from 5 to 10 and 20 parts per hundred part (pph) of Sm_2_O_3_. The outcomes of this work should not only reveal the theoretical effectiveness of Sm_2_O_3_ to attenuate thermal neutrons and self-emitted gamma rays but also present an appropriate method to improve the particle distribution, wear resistance, and mechanical properties of Sm_2_O_3_/UHMWPE composites by the surface treatment of Sm_2_O_3_, using KBE903.

## 2. Experimental

### 2.1. Simulation of Neutron and Self-Emitted Gamma-Shielding Properties of Sm_2_O_3_/UHMWPE Composites

#### 2.1.1. Determination of µ_m_, µ, and HVL

The Monte Carlo code, PHITS (version 3.22) was used to determine the µ_m_, µ, and HVL values of Sm_2_O_3_/UHMWPE composites with varying Sm_2_O_3_ contents of 0–50 wt.%. The neutron energy used for the investigation was 0.025 eV and, due to the emission of gamma rays with energies of 0.334, 0.712, and 0.737 MeV after neutron absorption by Sm, self-emitted gamma-shielding properties at the mentioned energies were separately determined. To simulate shielding properties by using PHITS, the code was set up such that neutron and gamma beams with diameters of 1 mm were directed at the center of a sample having a surface area of 20 cm × 20 cm and thickness of 0.5 and 1.0 cm for neutron and gamma attenuations, respectively, to the minimize effects of any build-up factor. To detect all primary transmitted neutrons and gamma rays, the detector with 100% detection efficiency was set up at the center of the sample [24,25]. The results from PHITS were independently verified by the comparison with theoretical shielding results, using a web-based program, XCOM. The details of the procedure for using XCOM to find the µ_m_ values of the Sm_2_O_3_/UHMWPE composites can be found elsewhere [13].

To determine the values of µ_m_ and HVL for the UHMWPE composites, the obtained values of µ from PHITS were used to calculate the desired quantities, following Equations (2) and (3), respectively [26]:

(2) $$\mu_{m}=\frac{\mu }{\rho }$$

(3) $$\mathrm{HVL}=\frac{\ln\left(2\right)}{\mu }$$

where *ρ* is the density of the composites theoretically estimated by using Equation (4):

(4) $$\rho =\frac{100}{\frac{C_{\mathrm{UHMWPE}}}{\rho_{\mathrm{UHMWPE}}}+\frac{C_{{\mathrm{Sm}}_{2}O_{3}}}{\rho_{{\mathrm{Sm}}_{2}O_{3}}}}$$

where *ρ*_UHMWPE_ and *ρ*_Sm__2O__3_ are the densities of UHMWPE and Sm_2_O_3_, respectively (*ρ*_UHMWPE_ = 0.94 g/cm^3^ [27] and *ρ*_Sm__2O__3_ = 8.35 g/cm^3^ [28]), while *C*_UHMWPE_ and *C*_Sm__2O__3_ are the contents of the UHWMPE and Sm_2_O_3_ in the Sm_2_O_3_/UHMWPE composites, respectively. It should be noted that *C*_UHMWPE_ + *C*_Sm__2O__3_ = 100 wt.%. The calculated densities of the UHMWPE composites containing varying Sm_2_O_3_ contents following Equation (4) are shown in Table 1.

To obtain the values of µ_m_, µ, and HVL for all Sm_2_O_3_ contents in the UHWMPE composites, as well as for the determination of the recommended Sm_2_O_3_ content in a later investigation, mathematical relationships between the shielding parameters and Sm_2_O_3_ contents were developed, for which the relationship between µ_m_ and the Sm_2_O_3_ content was represented in a linear form (Equation (5)), while the relationship between µ (HVL) and the Sm_2_O_3_ content was represented in an exponential form (Equation (6)):

(5) $$\mu_{m}=Ax+B$$

(6) $$\mu \;\left(\mathrm{HVL}\right)={\mathrm{Ae}}^{Bx}$$

where *x* is the Sm_2_O_3_ content and A (B) is the mathematical constant, determined by using a trendline function available in the Microsoft Excel software package.

#### 2.1.2. Determination of Recommended Sm_2_O_3_ Content

To determine the recommended Sm_2_O_3_ content, the values of µ_m_, µ, and HVL for the PE composites containing 5 wt.% boron, which was a commercially common neutron-shielding material used in general nuclear facilities [29], were determined by using PHITS, following the same setup and procedure as those for the Sm_2_O_3_/UHMWPE composites. Then, the obtained µ_m_, µ, and HVL values of the referenced material were inserted into the mathematical relationships from Section 2.1.1 (Equations (5) and (6)) to obtain the values of x, which could be regarded as the recommended Sm_2_O_3_ content in the UHMWPE composites for actual use that exhibited equal neutron-shielding abilities to the commercial ones.

### 2.2. Determination of Effects of Silane Surface Treatment on Sm_2_O_3_/UHMWPE Composites

#### 2.2.1. Materials and Chemicals

UHMWPE powder (U310 grade), which was used as the main matrix, was obtained from IRPC Public Co., Ltd. (Bangkok, Thailand). The average molecular mass, the density, and the average particle size of UHMWPE were 3.5 × 10^6^ g/mol, 0.94 g/cm^3^, and 170 µm, respectively. The formulations, including all the chemicals, their roles, and their suppliers, are given in Table 2. It should be noted that paraffinic oil was used in this work to overcome the ultrahigh viscosity of UHMWPE, which has an intrinsic viscosity of 1700 mL/g, during the twin-screw extrusion process used for compounding. Furthermore, to perform surface treatment on the Sm_2_O_3_ particles before the usual sample preparation, a silane coupling agent (3-aminopropyltriethoxysilane; KBE903) and other chemicals were used; their names, contents, and suppliers are given in Table 3.

#### 2.2.2. Silane Surface Treatment of Sm_2_O_3_ Particles

A silane coupling agent (KBE903), 99% ethanol, and distilled water, with their contents as shown in Table 3, were mixed and continuously stirred, using a magnetic stirrer (Phoenix, RSM-01, Garbsen, Germany) for 15 min. Then, Sm_2_O_3_ particles were added to the mixture and the stirring continued for another 30 min at a rotation speed of 1000 rpm. The mixture was transferred to a water bath and stirred at a water temperature of 90 °C for 1 h. Lastly, the mixture was oven-dried at 80 °C for 3 h to completely evaporate any ethanol and distilled water.

#### 2.2.3. Preparation of Sm_2_O_3_/UHMWPE Composites

UHMWPE, treated Sm_2_O_3_, and paraffinic oil were mixed by using a high-speed mixer (LMXS, Lab Tech Engineering, Bangkok, Thailand) following the formulation shown in Table 2. The mixture was then compounded by using a twin-screw extruder (CTW 1000C, Haake Rheomax, Germany) with temperature settings of 170, 175, 180, and 185 °C for the feed zone, plastification zone, mixing zone, and die zone, respectively. The speed used for the twin-screw extrusion was set at 40 rpm. The extrudate was pelletized into granules and soaked in n-hexane for 24 h to dissolve and extract any paraffinic oil from the granules. Then, the extrudate was put in a hot-air oven (GT-7017-L, Gotech Testing Machine, Taichung City, Taiwan) at 80 °C for 12 h for further extraction of the remaining n-hexane. The samples of the Sm_2_O_3_/UHMWPE composites were formed by molding the granules, using a hot press (LP-20M LMXS, Lab Tech Engineering, Bangkok, Thailand) at a pressure of 15 MPa and a temperature of 185 °C, for 14 min. It should be noted that a Sm_2_O_3_ content of 25 wt.% was selected for this investigation based on the results from our previous report, which showed that the Sm_2_O_3_/UHMWPE composites exhibited relatively constant shielding properties at high Sm_2_O_3_ contents, starting at around 20–30 wt.%. Hence, it was reasonable to carry out the investigation on the effects of silane surface treatment at 25 wt.% filler content [14].

#### 2.2.4. Characterization

##### Wear and Frictional Properties

The wear and frictional properties of the Sm_2_O_3_/UHMWPE composites were determined, using a ball-on-disc tester, with an applied normal load of 5 N, a constant sliding speed of 0.3 m/s, and a sliding distance of 1000 m. The specimens were cut into a square with a dimension of 40 mm × 40 mm and 4 mm in thickness, while a high-chromium steel ball (SKF, Bangkok, Thailand) with a diameter of 6 mm was used as the counterpart. The specific wear rate (*W_sp_*) for each sample was calculated by using Equation (8):

(7) $$W_{\mathrm{sp}}=\frac{V}{L\times D}$$

where *W_sp_*, *V*, *L*, and *D* are the specific wear rate, the total wear volume, the normal load, and the total sliding distance, respectively [30]. Furthermore, the surface roughness of the Sm_2_O_3_/UHMWPE composites after the measurement of wear rates was also determined by using a surface-roughness tester (SURFTEST SV-3100, Mitutoyo, Kawasaki, Japan) with a testing speed of 100 µm/s.

##### Mechanical Properties

The tensile properties (tensile modulus, tensile strength, and elongation at break) were determined by using a universal testing machine (Autograph AG-I 5 kN, Shimadzu, Kyoto, Japan) according to ASTM D638-14 standard testing. The specimens were cut into a dumbbell shape (Type I) with a thickness of 2 mm and the tensile speed used for all measurement was 100 mm/min. For surface hardness measurement, all specimens were tested by using a hardness durometer (Shore D) (Techlock GS-719G, Nagano, Japan), following ASTM D2240-03 standard testing.

##### Particle Sizes, Elemental Compositions, and Morphological Properties

The particle sizes of the non-treated and treated Sm_2_O_3_ particles, as well as the elemental composition and morphological properties of Sm_2_O_3_/UHMWPE composites were determined by using a scanning electron microscope with energy dispersive X-ray spectrometer (SEM–EDX) (TM4000PLUSII, Hitachi, Japan) at a 10 kV accelerating voltage. The average particle sizes of the Sm_2_O_3_ particles were determined by using micrographs from the SEM and the ImageJ software version 1.50i, which showed the average particle size was 15.3± 5.3 µm for the non-treated Sm_2_O_3_.

##### X-ray Diffraction (XRD) and Fourier-Transform Infrared Spectroscopy (FTIR)

The crystallinity of the Sm_2_O_3_/UHMWPE composites was determined by using an XRD technique (Bruker D8 Advance, Bruker, San Jose, USA). The XRD source was Ni-filtered CuK_α_ radiation with scanning angles (2θ) in the range 5–50° and a scanning speed of 0.02°/step. The active functional groups of the non-treated and treated Sm_2_O_3_ particles were determined by using FTIR (Vertex 70, Bruker, San Jose, USA), with wavenumbers in the range 400–4000 cm^−1^.

## 3. Results and Discussion

### 3.1. Simulated Neutron and Self-Emitted Gamma-Shielding Properties

The simulated values of µ, HVL, and µ_m_ for neutron and self-emitted gamma attenuation of the Sm_2_O_3_/UHMWPE composites, with varying Sm_2_O_3_ contents of 0–50 wt.%, are shown in Table 4, Table 5 and Table 6, respectively. The results indicated that the addition of Sm_2_O_3_ enhanced the abilities to attenuate thermal neutrons and gamma rays, as evidenced by the increases in µ and µ_m_, and the decrease in HVL. Furthermore, the results revealed that the gamma-shielding properties of the composites decreased with increasing gamma energies, as seen by the lower µ and µ_m_ values and the higher HVL values for samples determined at a gamma energy of 0.737 MeV compared to those at gamma energies of 0.712 and 0.334 MeV, respectively. Furthermore, the correctness of the results from PHITS were verified by comparison with those obtained from XCOM, with good agreement obtained between the two methods, as shown by the range and average percentage differences being 0.24–1.75% and 0.92%, respectively (Supplementary Materials Table S1), implying the reliability of the results from PHITS for further investigation.

**Figure 1:**
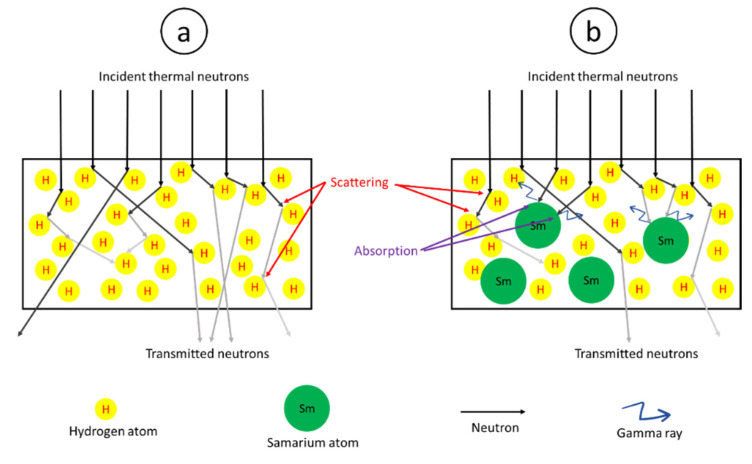
Scheme showing mechanisms of thermal neutron attenuation of (**a**) neat UHMWPE and (**b**) Sm_2_O_3_/UHMWPE composite.

Enhancement in the abilities to attenuate thermal neutrons in the UHMWPE composites after the addition of Sm_2_O_3_ was due to the increased probability of interactions through neutron absorption from the added Sm_2_O_3_, for which the efficiency of neutron attenuation through absorption is considerably higher than through scattering that requires multiple collisions between the incident neutrons and the materials before their energies become negligible. It should be noted that the scattering cross-section (σ_s_) and the absorption cross-section (σ_a_) for H are 82.02 and 0.3326 barns, respectively, while σ_s_ and σ_a_ for Sm are 39.3 and 5922 barns, respectively [31], making Sm and Sm-containing compounds more superior neutron attenuators than H and H-containing compounds. The schemes graphically illustrating effects of fillers (Sm_2_O_3_ in this work) on neutron attenuations through the mechanism of scattering and absorption are shown in Figure 1; with Figure 1a representing the attenuation mechanisms of a neat UHMWPE through scattering and Figure 1b representing the attenuation mechanisms of Sm_2_O_3_/UHMWPE composites through both scattering and absorption, making the latter more efficient in reducing the numbers of transmitted neutrons [13].

On the other hand, enhancement in the abilities to attenuate self-emitted gamma rays after the addition of Sm_2_O_3_ was mainly due to the relatively high atomic number (Z) and density (*ρ*) of Sm and Sm_2_O_3_, respectively, compared to those for C and H in a neat UHMWPE. The high values of Z and *ρ* of Sm_2_O_3_ led to higher interaction probabilities between the incident gamma rays and the composites through photoelectric absorption and Compton scattering, resulting in substantially lesser gamma transmission. It was notable that the photoelectric cross-section (σ_pe_) and Compton scattering cross-section (σ_comp_) were related to gamma energies and material characteristics as shown in Equations (8) and (9), respectively [32]:

(8) $$\sigma_{\mathrm{pe}}\propto \frac{Z^{n}}{{\left(h\nu \right)}^{3}}$$

(9) $$\sigma_{\mathrm{comp}}\propto \frac{1}{n_{e}}$$

where Z is the atomic number of the target, h is Planck’s constant, ν is the frequency that directly relates to the gamma energy (E = hν), and n_e_ is the electron density of the target. As Equation (9) depicts, σ_pe_, which is a dominant interaction at low gamma energies, is directly proportional to Z^n^ and inversely proportional to (hν)^3^ or E^3^, resulting in higher µ values for the composites containing heavy elements (high Z) and lower µ values at higher gamma energies, respectively.

To determine the recommended Sm_2_O_3_ content that exhibited equal neutron-shielding abilities to the commercial 5% borated PE, mathematical relationships between neutron-shielding parameters (µ, HVL, and µ_m_) and the Sm_2_O_3_ contents (their forms shown in Equations (5) and (6)) are shown in Table 7. Since the simulated µ, HVL, and µ_m_ values of 5% borated PE, determined by using PHITS, were 4.8062 cm^−1^, 0.1442 cm, and 5.0592 cm^2^/g, respectively (Table 4, Table 5 and Table 6, respectively), the recommended range in the Sm_2_O_3_ content of 11–13 wt.% made the UHMWPE composites acceptable for use in general nuclear facilities. This recommended Sm_2_O_3_ content range was actually lower than the required B_2_O_3_ contents of 15.9 wt.% that provides 5% of B in the composites, resulting in less filler added and possibly better preserved preferred properties of UHMWPE. In addition to the superior neutron-shielding properties of the Sm_2_O_3_/UHMWPE composites compared to 5% borated PE, it was clear from Table 4, Table 5 and Table 6 that the former exhibited better gamma attenuation than the latter, implying better overall shielding properties of the Sm_2_O_3_/UHMWPE composites for both thermal neutrons and gamma rays.

### 3.2. Effects of Silane Surface Treatment on Sm_2_O_3_ Particles

**Figure 2:**
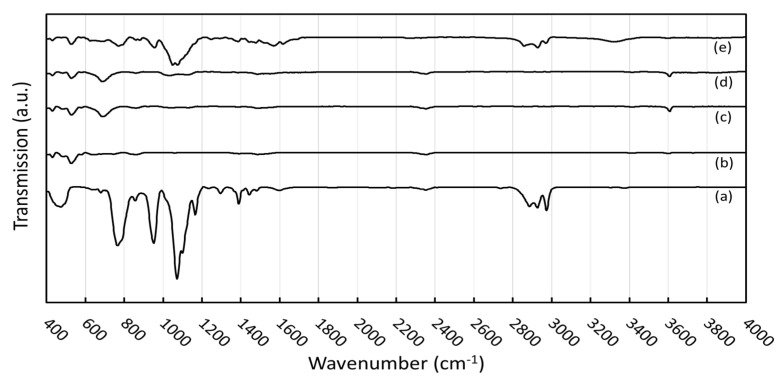
FTIR spectra of (**a**) silane coupling agent (KBE903), (**b**) non-treated Sm_2_O_3_, (**c**) treated Sm_2_O_3_ with 5 pph KBE903, (**d**) treated Sm_2_O_3_ with 10 pph KBE903, and (**e**) treated Sm_2_O_3_ with 20 pph KBE903.

**Figure 3:**
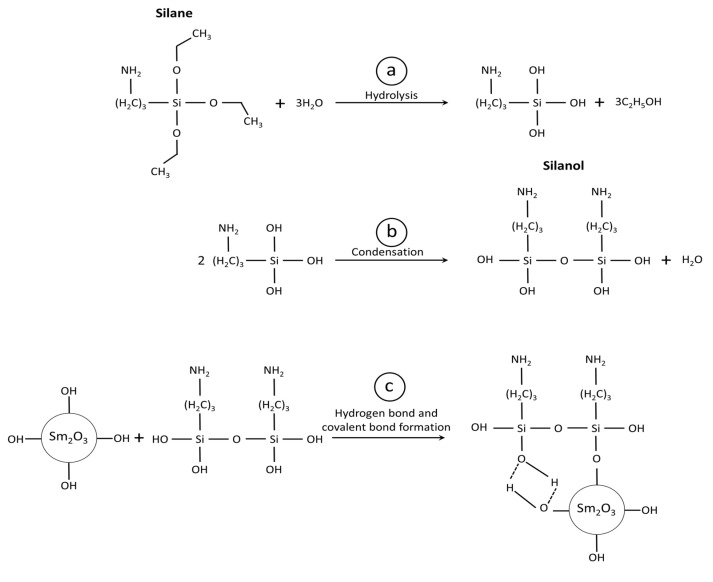
Schemes showing mechanisms of grafting KBE903 on surface of Sm_2_O_3_, including (**a**) hydrolysis, (**b**) condensation, and (**c**) hydrogen bond and covalent bond formation.

The FTIR spectra of the silane coupling agent (KBE903), non-treated Sm_2_O_3_, and treated Sm_2_O_3_ are shown in Figure 2. For the spectra of KBE903, the main peaks were observed at 476, 767, 954–1074, 1400–1500, and 2851–2923 cm^−1^, which corresponded to Si–O–Si, Si–O–C, Si–O, N–H, and C–H, respectively [33]. The spectra of the non-treated Sm_2_O_3_ showed broad peaks at around 530 cm^−1^, which corresponded to Sm_2_O_3_ stretching vibration. In the spectra of the treated Sm_2_O_3_, the additional peaks observed at 698, 960–1074, and 3608–3610 cm^−1^ corresponded to Si–C stretching vibration, Si–O stretching vibration, and –OH stretching vibration from water absorption, respectively. The presence of Si–O–C and Si–O in the treated Sm_2_O_3_ evidently indicated the successful treatment of Sm_2_O_3_ particles by grafting KBE903 on their surfaces [34]. It was noteworthy that the treated Sm_2_O_3_ with 20 pph KBE903 exhibited a higher footprint of KBE903 than those with 5 pph and 10 pph, probably due to the excessive use of KBE903 during the surface treatment that led to higher amounts of KBE903 remaining in the filler. The schemes showing the mechanism of grafting KBE903 on the surface of Sm_2_O_3_ are shown in Figure 3, which includes three main steps, namely hydrolysis, condensation, and the formation of hydrogen and covalent bonds [35].

The average particle sizes of Sm_2_O_3_ for each treatment were determined by using the ImageJ software. The results revealed that the Sm_2_O_3_ particles with 20 pph KBE903 had the largest average particle size as well as the largest deviation in particle sizes of 21.5 ± 5.3 µm compared to the non-treated Sm_2_O_3_ (15.3 ± 5.3 µm) and treated Sm_2_O_3_ at 5 pph and 10 pph KBE903 (14.3 ± 2.9 µm and 11.7 ± 2.4 µm, respectively). This behavior was observed because, although the application of KBE903 at low contents (5 and 10 pph in this work) could limit/reduce particle agglomeration after the treatment, excessive use of KBE903 would instead worsen the agglomeration of Sm_2_O_3_ particles from the increase in self-condensation reactions, leading to larger average particle sizes and deviations in the samples with 20 pph KBE903 [36].

### 3.3. Elemental Composition of Treated Sm_2_O_3_ Particles and Treated Sm_2_O_3_/UHMWPE Composites

The elemental compositions of treated Sm_2_O_3_ particles and treated Sm_2_O_3_/UHMWPE composites, determined by using SEM–EDX, are shown in Table 8. The results indicated that the treated Sm_2_O_3_ particles mainly composed of O, Sm, and Si elements, with small deviations among each silane content (the differences were within their standard deviations). Similarly, the treated Sm_2_O_3_/UHMWPE composites mainly composed of C, O, Sm, and Si elements, also with small deviations among each silane content. These results implied that different conditions of silane surface treatment did not substantially alter elemental compositions of the fillers and the composites. Hence, any observed changes in the properties investigated in this work were due to effects of silane on bonding/dispersing fillers in the UHMWPE matrix. It is noteworthy that the Si compositions for treated Sm_2_O_3_ particles and treated Sm_2_O_3_/UHMWPE composites with 20 pph KBE903 were noticeably higher than those with 5 and 10 pph KBE903, due to the higher use of KBE903 that resulted in higher Si compositions remaining in the Sm_2_O_3_ particles and Sm_2_O_3_/UHMWPE composites.

### 3.4. Wear, Frictional, and Mechanical Properties of Treated Sm_2_O_3_/UHMWPE Composites

**Figure 4:**
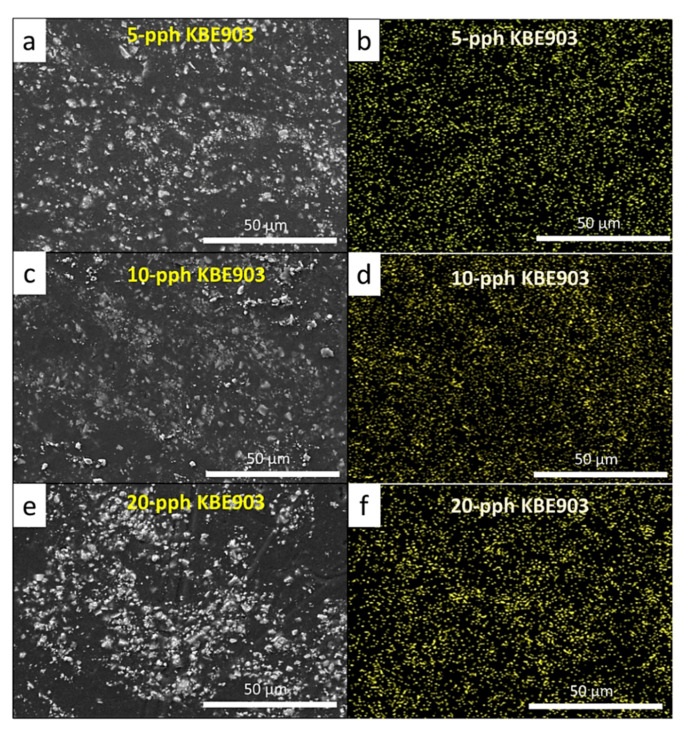
SEM and EDX images of treated Sm_2_O_3_/UHMWPE composites, where (**a**,**b**) are those with 5 pph KBE903, (**c**,**d**) are those with 10 pph KBE903, and (**e**,**f**) are those with 20 pph KBE903. The yellow dots in (**b**,**d**,**f**) represent Sm elements in composites. All images are at a magnification of ×1000.

The specific wear rates (*W_sp_*), average coefficient of friction, surface roughness, and crystallinity of the treated Sm_2_O_3_/UHMWPE composites with varying silane contents of 5, 10, and 20 pph and a fixed Sm_2_O_3_ content of 25 wt.% are shown in Table 9. The results indicated that *W_sp_* of the treated Sm_2_O_3_ composites, especially the sample with 5 pph KBE903, provided lower *W_sp_* values in comparison with the non-treated samples, which had the value of 5.69 × 10^−5^ mm^3^/Nm. The reduction in *W_sp_* for treated Sm_2_O_3_/UHMWPE composites, which indicated an enhancement in their wear resistance, were due to the formation of Si-O bonds by the KBE903 molecules that improved the dispersion of the Sm_2_O_3_ particles (Figure 4), as well as strengthened interfacial-bonding between Sm_2_O_3_ and the UHMWPE matrix [37].

**Figure 5:**
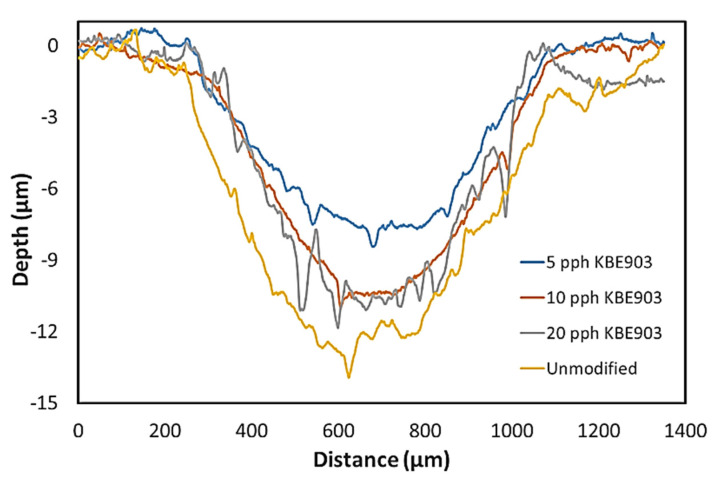
Worn surface profiles of non-treated (unmodified) and treated Sm_2_O_3_/UHMWPE composites.

In addition, Table 9 showed that the percentages of crystallinity for all treated samples were greater than 70%, while the non-treated samples exhibited lower crystallinity of 66.83 ± 7.72%. This enhanced crystallinity in treated samples could have been due to the increased intermolecular bonding between Sm_2_O_3_ and UHMWPE from the application of KBE903 that reduced the mobility of the chain segments of the UHMWPE network and, hence, increased crystallinity and wear resistance of the composites [38]. However, as the KBE903 content further increased from 5 pph to 10 and 20 pph, the value of *W_sp_* started to increase again. This could have been because of the high application of KBE903, especially for those with 20 pph KBE903, that led to higher agglomeration of Sm_2_O_3_ particles (Figure 4e,f) from the increase in self-condensation reactions of the silane coupling agent [39] and weaker interfacial bonding between Sm_2_O_3_ and the UHMWPE matrix that led to greater removal of Sm_2_O_3_ lumps during the frictional process (higher *W_sp_*) and higher surface roughness found in the sample with 20 pph KBE903 [36]. Furthermore, Figure 5 shows wear-depth profiles of the UHMWPE composites that were determined by using a stylus profilometer. The results indicated that higher wear depths were found in non-treated (unmodified) samples in comparison with treated samples, which could be explained by weaker bonding between non-treated Sm_2_O_3_ particles and the UHMWPE matrix that led to greater wear depths and, subsequently, larger *W_sp_*.

**Figure 6:**
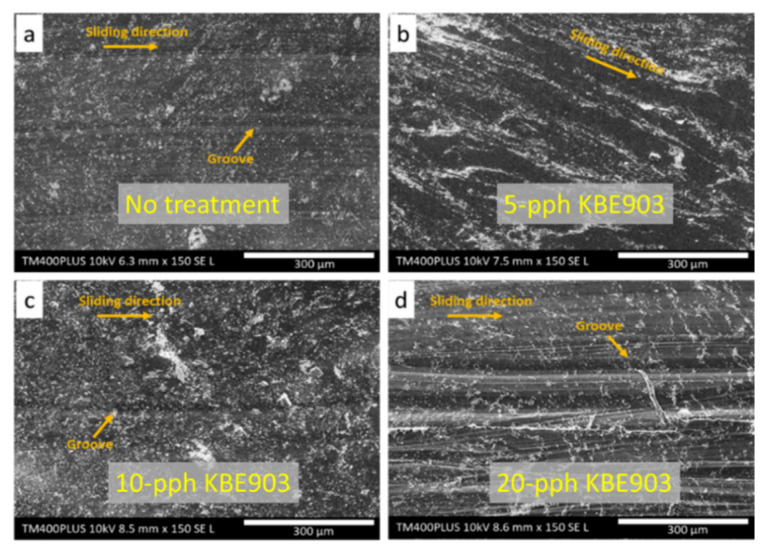
SEM images showing wear tracks of (**a**) non-treated Sm_2_O_3_/UHMWPE composites and treated Sm_2_O_3_/UHMWPE composites with (**b**) 5 pph KBE903, (**c**) 10 pph KBE903, and (**d**) 20 pph KBE903. All images are at a magnification of ×150.

Figure 6 illustrates the micrographs of wear tracks for the UHMWPE composites after the dry sliding test on the pin-on-disc tester. The micrographs revealed that wear tracks of samples with 5 pph KBE903 were smoother than those of other treatment conditions, probably due to effects of the dominant adhesive wear mechanism that created a plastic deformation on the worn surfaces and subsequently smoother wear tracks (Figure 6b). On the other hand, wear tracks of non-treated and treated samples with 10 and 20 pph KBE903 showed rough grooves and lump debris in their wear tracks (Figure 6a,c,d). These behaviors were observed due to higher particle agglomeration and greater removal of Sm_2_O_3_ lumps during the dry sliding test, caused by the combined two- and three-body abrasive mechanisms [30,40]; that is, the harder counterface of a high-chromium steel ball resulted in a two-body abrasive mechanism, while the granular debris trapped between the counterface resulted in a three-body abrasive mechanism, which subsequently resulted in rougher wear tracks in the sample with 10 and 20 pph KBE903. The results from wear track analysis clearly confirmed and supported the results of the wear resistance, which saw the samples with lower KBE903 contents having relatively lower *W_sp_* than those without the surface treatment and those with 20 pph KBE903.

**Figure 7:**
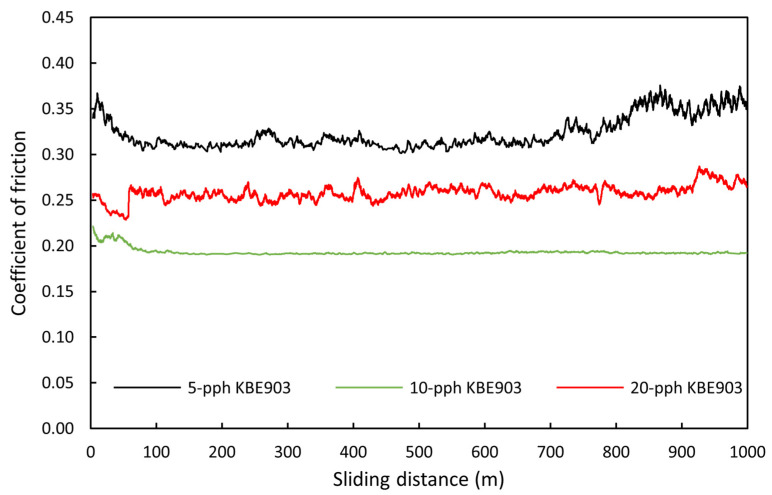
Coefficient of friction with respect to sliding distance of treated Sm_2_O_3_/UHMWPE composites with varying silane contents of 5, 10, and 20 pph and Sm_2_O_3_ content of 25 wt.%.

Furthermore, the investigation on frictional properties revealed that the treated Sm_2_O_3_/UHMWPE composites with 10 pph KBE903 exhibited the lowest coefficient of friction and the least fluctuation in its values with respect to sliding distance compared to other conditions, as shown in Figure 7. This could have been because loose Sm_2_O_3_ particles and wear debris from Sm_2_O_3_/UHMWPE composites occurred during the dry sliding test were trapped between the surfaces, resulting in lesser forces required to move and roll the ball during the dry sliding test [9,41]. It should be noted that, although Sm_2_O_3_/UHMWPE composites with 5 pph KBE903 exhibited the lowest *W_sp_*, their coefficient of friction was higher than other treatment conditions. This was mainly due to the results from the dominant adhesive wear mechanism that created plastic deformation on their worn surfaces (Figure 6b). On the other hand, the coefficient of friction for Sm_2_O_3_/UHMWPE composites with 20 pph KBE903 exhibited a higher value than those from the sample with 10 pph KBE903. This was because the excessive use of 20 pph KBE903 reduced the uniformity of Sm_2_O_3_ dispersion that resulted in more pronounced ploughing tracks on the worn surfaces from the abrasive wear mechanism and subsequently higher coefficients of friction.

The tensile properties (tensile modulus, tensile strength, and elongation at break) and surface hardness (Shore D) of the treated Sm_2_O_3_/UHMWPE composites with varying KBE903 contents of 5, 10, and 20 pph are shown in Table 10. The results indicated that the samples with the surface treatment of 10 pph KBE903 had the highest overall tensile properties among all investigated conditions. These results were observed because, similar to the wear properties, the utilization of 10 pph KBE903 for the surface treatment of Sm_2_O_3_ was able to disperse the Sm_2_O_3_ particles in the UHMWPE composites (Figure 4) and to form strong interfacial bonding between the Sm_2_O_3_ particles and the UHMWPE matrix, leading to higher overall tensile properties. In addition, due to the high rigidity of Sm_2_O_3_ and the more uniform distribution of Sm_2_O_3_ inside the matrix, the surface hardness (Shore D) of the sample from the 10 pph KBE903 treatment also had the highest value compared to those with the other KBE903 contents. It should be noted that, since all UHWMPE composites in this work had the surface hardness (Shore D) values greater than 62, the composites were considered to be extra-hard materials [42]. Hence, indentation depth from the surface hardness (Shore D) measurement, which used a durometer (Teclock GS-719G, Japan) having an indenter shape of a truncated cone with a 0.79 mm diameter and 2.50 mm height [43], was barely observed, implying the high resistance of their surfaces to deform under external force.

To emphasize the advantage of performing a silane surface treatment, especially with KBE903, the tensile strength of the treated Sm_2_O_3_/UHMWPE composites with 10 pph KBE903 was compared with the results for the non-treated Sm_2_O_3_/UHMWPE composites from our previous report, with the former having a higher tensile strength than the latter that had the tensile strength of approximately 21.7 MPa (interpolated from our previous work, which reported the tensile strengths of 23.1 and 20.2 MPa for UHMWPE composites containing 20 and 30 wt.% of non-treated Sm_2_O_3_, respectively [14]). It should be noted that the samples from this work and the non-treated Sm_2_O_3_/UHMWPE composites from our previous report were prepared by using the same setup and procedure.

## 4. Conclusions

This work determined the thermal neutron and self-emitted gamma attenuations of UHMWPE composites containing varying Sm_2_O_3_ contents of 0–50 wt.% at a neutron energy of 0.025 eV and varying gamma energies of 0.334, 0.712, and 0.737 MeV, using a simulation code, namely PHITS. The current work also investigated the effects of a silane coupling agent (namely KBE903, used for the surface treatment of Sm_2_O_3_ particles) on the morphological, wear, frictional, and mechanical properties of UHMWPE composites containing 25 wt.% of Sm_2_O_3_. The results from the PHITS simulation indicated that the abilities to attenuate thermal neutrons and self-emitted gamma rays of the composites increased with increasing Sm_2_O_3_ contents, while the gamma-shielding properties of the composites tended to decrease with increasing gamma energies (determined at the same filler content), as seen as by the higher values of µ and µ_m_ at a gamma energy of 0.334 MeV compared to those at gamma energies of 0.712 and 0.737 MeV, respectively. The comparison of the thermal neutron-shielding properties of the Sm_2_O_3_/UHMWPE composites with a commercial material containing 5% boron indicated that the composites with Sm_2_O_3_ contents of 11–13 wt.% could attenuate thermal neutrons with equal efficiency as the commercial product. In addition, the surface treatment of Sm_2_O_3_ that used 5–10 pph KBE903 resulted in enhanced wear resistance and tensile properties, as compared to those of non-treated Sm_2_O_3_/UHMWPE composites and treated Sm_2_O_3_/UHMWPE composites with 20 pph KBE903. The overall outcomes of this work suggested the great potential of utilizing Sm_2_O_3_ as a superior neutron attenuator in UHMWPE composites, with a preferred dual-shielding property. Furthermore, the surface treatment of Sm_2_O_3_ with 5–10 pph KBE903 could successfully enhance the wear resistance and strength of the composites, increasing the usability and processability of the product.

## Figures and Tables

**Table 1:** Theoretical densities of UHMWPE composites containing varying Sm_2_O_3_ contents in the range 0–50 wt.%.

Sm_2_O_3_ Content (wt.%)	Density (g/cm^3^)
0	0.940
5	0.994
10	1.042
15	1.096
20	1.155
25	1.220
30	1.294
35	1.377
40	1.472
45	1.580
50	1.706

**Table 2:** Material formulations of UHMWPE composites and their chemicals, roles, and suppliers.

Chemical	Content (wt.%)	Function	Supplier
UHMWPE	75	Main matrix	IRPC Public Co., Ltd. (Bangkok, Thailand)
Samarium oxide (Sm_2_O_3_)	25	Radiation protective filler	Richest Group (Shanghai, China)
Paraffinic oil *	10% of UHMWPE	Processing aid	Facobis Co., Ltd. (Bangkok, Thailand)

* Paraffinic oil was later extracted by using n-hexane after the extrusion process.

**Table 3:** Formulations for surface treatment of Sm_2_O_3_ particles and their suppliers.

Chemical	Content (pph)	Supplier
Samarium oxide (Sm_2_O_3_)	100	Richest Group (Shanghai, China)
Silane coupling agent (KBE903)	5, 10, and 20	Kisco(T) Ltd. (Bangkok, Thailand)
99% Ethanol	92 − x *	Gammaco Co., Ltd. (Bangkok, Thailand)
Distilled water	8	Kasetsart University (Bangkok, Thailand)

* x is the content of KBE903.

**Table 4:** Linear attenuation coefficients (µ) for neutron and self-emitted gamma attenuation of Sm_2_O_3_/UHMWPE composites and a commercial 5% borated PE, determined by using PHITS.

Filler	Filler Content (wt.%)	Linear Attenuation Coefficient (cm^−1^)			
		Thermal Neutron	Gamma Rays		
			0.334 MeV	0.712 MeV	0.737 MeV
Sm	0	2.7025	0.1129	0.0819	0.0805
	5	3.6516	0.1206	0.0852	0.0838
	10	4.7053	0.1296	0.0894	0.0877
	15	5.9034	0.1403	0.0940	0.0924
	20	7.1626	0.1503	0.0977	0.0961
	25	8.6581	0.1632	0.1031	0.1011
	30	10.2055	0.1765	0.1087	0.1066
	35	12.0982	0.1924	0.1155	0.1133
	40	14.0712	0.2098	0.1227	0.1202
	45	16.2235	0.2298	0.1310	0.1282
	50	19.1070	0.2543	0.1409	0.1381
B	5	4.8062	0.1162	0.0848	0.0833

**Table 5:** Half-value layer (HVL) for neutron and self-emitted gamma attenuation of Sm_2_O_3_/UHMWPE composites and a commercial 5% borated PE, determined by using PHITS.

Filler	Filler Content (wt.%)	Half-Value Layer (cm)			
		Thermal Neutron	Gamma Rays		
			0.334 MeV	0.712 MeV	0.737 MeV
Sm	0	0.2565	6.1419	8.4671	8.6140
	5	0.1898	5.7481	8.1378	8.2705
	10	0.1473	5.3486	7.7539	7.8995
	15	0.1174	4.9389	7.3760	7.5054
	20	0.0968	4.6129	7.0941	7.2142
	25	0.0801	4.2473	6.7214	6.8531
	30	0.0679	3.9274	6.3738	6.5014
	35	0.0573	3.6035	6.0033	6.1171
	40	0.0493	3.3045	5.6500	5.7667
	45	0.0427	3.0159	5.2902	5.4082
	50	0.0363	2.7259	4.9186	5.0189
B	5	0.1442	5.9632	8.1772	8.3248

**Table 6:** Mass attenuation coefficients (µ_m_) for neutron and self-emitted gamma attenuation of Sm_2_O_3_/UHMWPE composites and a commercial 5% borated PE, determined by using PHITS.

Filler	Filler Content (wt.%)	Mass Attenuation Coefficient (cm^2^/g)			
		Thermal Neutron	Gamma Rays		
			0.334 MeV	0.712 MeV	0.737 MeV
Sm	0	2.8447	0.1187	0.0861	0.0847
	5	3.6735	0.1213	0.0856	0.0843
	10	4.5140	0.1243	0.0857	0.0841
	15	5.3880	0.1280	0.0857	0.0842
	20	6.2032	0.1301	0.0846	0.0832
	25	7.0946	0.1337	0.0845	0.0828
	30	7.8865	0.1363	0.0840	0.0823
	35	8.7848	0.1396	0.0838	0.0822
	40	9.5611	0.1425	0.0833	0.0816
	45	10.2668	0.1454	0.0829	0.0811
	50	11.2005	0.1490	0.0821	0.0809
B	5	5.0592	0.1223	0.0892	0.0876

**Table 7:** Mathematical relationships between neutron-shielding parameters (µ, HVL, and µ_m_) and Sm_2_O_3_ contents and their corresponding mathematical coefficients (A and B).

Quantity	Mathematical Form	Mathematical Coefficient	
		A	B
µ	$\mu ={\mathrm{Ae}}^{\mathrm{Bx}}$	3.1232	0.0379
HVL	$\mathrm{HVL}={\mathrm{Ae}}^{\mathrm{Bx}}$	0.2219	−0.038
µ_m_	$\mu_{m}=A$x + B	0.1669	2.8666

**Table 8:** Elemental composition of treated Sm_2_O_3_ particles and treated Sm_2_O_3_/UHMWPE composites.

Element	Elemental Composition (by Weight) (%)					
	Treated Sm_2_O_3_ Particles			Treated Sm_2_O_3_/UHMWPE Composites		
	Silane Content (pph)					
	5	10	20	5	10	20
C	-	-	-	69.69 ± 1.55	68.20 ± 1.72	67.85 ± 1.71
O	25.77 ± 1.89	26.69 ± 0.16	24.90 ± 2.19	6.78 ± 0.38	7.43 ± 0.67	7.43 ± 0.92
Sm	73.76 ± 2.52	72.83 ± 2.42	74.34 ± 2.11	23.21 ± 1.33	23.81 ± 1.14	24.04 ± 0.69
Si	0.47 ± 0.20	0.48 ± 0.05	0.75 ± 0.10	0.33 ± 0.03	0.55 ± 0.08	0.67 ± 0.10

**Table 9:** Specific wear rate, average coefficient of friction, surface roughness, and crystallinity of treated Sm_2_O_3_/UHMWPE composites with varying silane contents of 5, 10, and 20 pph and Sm_2_O_3_ content of 25 wt.%.

Silane Content (pph)	Specific Wear Rate (×10^−5^ mm^3^/Nm)	Average Coefficient of Friction	Roughness (µm)	Crystallinity (%)
5	1.78	0.32 ± 0.03	0.27 ± 0.05	71.10 ± 6.24
10	4.17	0.19 ± 0.01	0.20 ± 0.03	86.73 ± 2.06
20	4.25	0.25 ± 0.03	0.31 ± 0.14	78.87 ± 2.80

**Table 10:** Tensile properties of treated Sm_2_O_3_/UHMWPE composites with varying silane contents of 5, 10, and 20 pph.

Silane Content (wt.%)	Tensile Modulus (MPa)	Tensile Strength (MPa)	Elongation at Break (%)	Hardness (Shore D)
5	136.7 ± 28.4	22.0 ± 1.5	364 ± 42	63 ± 1
10	156.8 ± 20.9	24.9 ± 0.6	497 ± 27	68 ± 1
20	158.8 ± 35.2	21.1 ± 0.8	361 ± 86	62 ± 1

## Data Availability

Not applicable.

## Supplementary Materials

The following are available online at https://www.mdpi.com/article/10.3390/polym13193390/s1. Table S1: Comparative mass attenuation coefficients (µ_m_) of Sm_2_O_3_/UHMWPE composites and their percentage of differences between the values determined from PHITS and XCOM at the gamma energies of 0.334, 0.712, and 0.737 MeV.
