# Supplementary material for: Simulation of Neutron/Self-Emitted Gamma Attenuation and Effects of Silane Surface Treatment on Mechanical and Wear Resistance Properties of Sm2O3/UHMWPE Composites
Source: Polymers (Basel). 2021 Oct 2;13(19):3390. doi: 10.3390/polym13193390 (PMC8512719; doi:10.3390/polym13193390)
Supplement: Supplementary file 1 [file polymers-13-03390-s001.zip › polymers-1388051-supplementary.pdf]

Supporting Information

Simulation of Neutron/Self-Emitted Gamma Attenuation and Effects of Silane Surface Treatment on Mechanical and Wear Resistance Properties of Sm_2O_3/UHMWPE Composites

Donruedee Toyen [1], Yupadee Paopun [1], Dararat Changjan [1], Ekachai Wimolmala [2], Sithipong Mahathanabodee [3], Theerasarn Pianpanit [4], Thitisorn Anekratmontree [4] and Kiadtisak Saenboonruang [4,5,*]

[1] Scientific Equipment and Research Division, Kasetsart University Research and Development Institute (KURDI), Kasetsart University, Bangkok 10900, Thailand; rdiddt@ku.ac.th (D.T.), rdiydp@ku.ac.th (Y.P.), rdidrc@ku.ac.th (D.C.);

[2] Polymer PROcessing and Flow (P-PROF) Research Group, Division of Materials Technology, School of Energy, Environment and Materials, King Mongkut's University of Technology Thonburi, Bangkok 10140, Thailand; ekachai.wim@kmutt.ac.th (E.W.);

[3] Department of Production Engineering, Faculty of Engineering, King Mongkut's University of Technology North Bangkok, Bangkok 10800, Thailand; sithipong.m@eng.kmutnb.ac.th (S.M.);

[4] Department of Applied Radiation and Isotopes, Faculty of Science, Kasetsart University, Bangkok 10900, Thailand; fscitap@ku.ac.th (T.P.), thitisorn.an@ku.th (T.A.);

[5] Specialized Center of Rubber and Polymer Materials in Agriculture and Industry (RPM), Faculty of Science, Kasetsart University, Bangkok 10900, Thailand

* Correspondence: kiadtisak.s@ku.th; Tel.: +662-562-5555 (ext. 646219)

Table S1. Comparative mass attenuation coefficients (μ_m) of Sm_2O_3/UHMWPE composites and their percentage of differences between the values determined from PHITS and XCOM at the gamma energies of 0.334, 0.712, and 0.737 MeV.

Sm_2O_3 content (wt%.)	0.334-MeV gamma rays			0.712-MeV gamma rays			0.737-MeV gamma rays		
	PHITS	XCOM	Difference (%)	PHITS	XCOM	Difference (%)	PHITS	XCOM	Difference (%)
0	0.1187	0.1169	1.52	0.0861	0.0852	1.05	0.0847	0.0839	0.94
5	0.1213	0.1200	1.07	0.0856	0.0849	0.82	0.0843	0.0835	0.95
10	0.1243	0.1231	0.97	0.0857	0.0846	1.28	0.0841	0.0832	1.07
15	0.1280	0.1263	1.33	0.0857	0.0842	1.75	0.0842	0.0828	1.66
20	0.1301	0.1294	0.54	0.0846	0.0839	0.83	0.0832	0.0824	0.96
25	0.1337	0.1325	0.90	0.0845	0.0836	1.07	0.0828	0.0820	0.97
30	0.1363	0.1356	0.51	0.0840	0.0832	0.95	0.0823	0.0817	0.73
35	0.1396	0.1388	0.57	0.0838	0.0829	1.07	0.0822	0.0813	1.09
40	0.1425	0.1419	0.42	0.0833	0.0826	0.84	0.0816	0.0809	0.86
45	0.1454	0.1450	0.28	0.0829	0.0822	0.84	0.0811	0.0805	0.74
50	0.1490	0.1482	0.54	0.0821	0.0819	0.24	0.0809	0.0817	0.99
